# Evaluation of Probiotics for Warfighter Health and Performance

**DOI:** 10.3389/fnut.2020.00070

**Published:** 2020-06-09

**Authors:** Richard T. Agans, Grace E. Giles, Michael S. Goodson, J. Philip Karl, Samantha Leyh, Karen L. Mumy, Kenneth Racicot, Jason W. Soares

**Affiliations:** ^1^Henry M. Jackson Foundation for the Advancement of Military Medicine, Bethesda, MD, United States; ^2^Naval Medical Research Unit Dayton, Environmental Health Effects Laboratory, Dayton, OH, United States; ^3^Soldier Performance Optimization Directorate, U.S. Army Combat Capabilities Development Command - Soldier Center, Natick, MA, United States; ^4^Air Force Research Laboratory, 711th Human Performance Wing, Wright Patterson Air Force Base, Dayton, OH, United States; ^5^Military Nutrition Division, U.S. Army Research Institute of Environmental Medicine, Natick, MA, United States; ^6^Oak Ridge Institute for Science and Education, Wright Patterson Air Force Base, Oak Ridge, TN, United States

**Keywords:** microbiota, probiotics, performance, cognition, warfighter, microbiome, physical, nutrition

## Abstract

The probiotic industry continues to grow in both usage and the diversity of products available. Scientific evidence supports clinical use of some probiotic strains for certain gastrointestinal indications. Although much less is known about the impact of probiotics in healthy populations, there is increasing consumer and scientific interest in using probiotics to promote physical and psychological health and performance. Military men and women are a unique healthy population that must maintain physical and psychological health in order to ensure mission success. In this narrative review, we examine the evidence regarding probiotics and candidate probiotics for physical and/or cognitive benefits in healthy adults within the context of potential applications for military personnel. The reviewed evidence suggests potential for certain strains to induce biophysiological changes that may offer physical and/or cognitive health and performance benefits in military populations. However, many knowledge gaps exist, effects on health and performance are generally not widespread among the strains examined, and beneficial findings are generally limited to single studies with small sample sizes. Multiple studies with the same strains and using similar endpoints are needed before definitive recommendations for use can be made. We conclude that, at present, there is not compelling scientific evidence to support the use of any particular probiotic(s) to promote physical or psychological performance in healthy military personnel. However, plausibility for physical and psychological health and performance benefits remains, and additional research is warranted. In particular, research in military cohorts would aid in assessing the value of probiotics for supporting physical and psychological health and performance under the unique demands required of these populations.

## Introduction

Health, readiness, and performance (defined as the ability to meet mission demands) are important measures within the military. The men and women who serve are held to stringent standards within each of those metrics throughout their military careers, ensuring that forces retain high capability (1–3). Military personnel are also often required to operate under conditions of sub-optimal sleep and/or nutrition, in extreme environments, and under elevated stress. In these situations, failure to perform optimally could mean the difference between mission success and failure. Some programs exist to promote healthy lifestyles, such as the Army's Performance Triad program. This program focuses on getting optimal sleep, activity, and nutrition in order to achieve the health and readiness goals required to ensure mission success. Nevertheless, the desire to optimize individual performance has been reported as a driving factor for service members to take dietary supplements, and significantly more military personnel are now using dietary supplements than the general population (69% compared to 50%, respectively) (4–7).

Live microorganisms are increasingly being included in dietary supplements resulting in a global industry currently valued at over $40 billion and forecasted to amass $64 billion in sales by 2023 (8). Although foods containing bacteria and/or their metabolites have long been recognized for their “health preserving” properties, interest in isolating and consuming certain bacteria began for researchers in the late twentieth century (9–11). At that time, the term “probiotic” was created. The definition of probiotic has evolved over time, with recent consensus settling on “live microorganisms that, when administered in adequate amounts, confer a health benefit on the host” (12). Inherent in this definition is that not all microorganisms can be considered as probiotic, and correct use of the term requires strain level identification, empirical evidence of health benefits in the target host, and the delivery of live microbes in adequate doses to elicit the health benefit.

**Figure 1:**
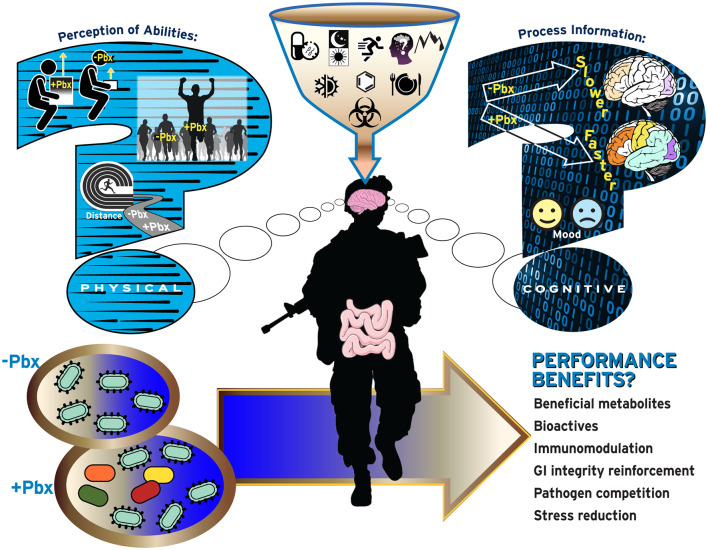
Current questions regarding probiotic use by military personnel. Increased use of probiotics (Pbx) may be perceived to increase physical abilities including increased muscle performance (top-left), physical performance (middle-left), or endurance performance (bottom-left), or cognitive performance related to information processing (top-right) and ability to handle emotions/mood (bottom-right).

The most common focus for probiotic research and development has been microbes that are administered orally to be delivered to the gastrointestinal (GI) tract. These ingested microbes compete and interact with the bacteria, archaea, viruses, and eukaryotes which constitute the commensal microbial content of the GI tract known as the “gut microbiome.” This research has led to the development of probiotics that have demonstrated efficacy in some populations suffering from upper respiratory tract infections (URTI), and GI-related maladies including travelers and acquired acute diarrhea, irritable bowel syndrome (IBS), inflammatory bowel disease (e.g., IBD, Crohn's disease, etc.), and lactose intolerance (10, 13–15). The putative mechanisms underlying health benefits of probiotics are not fully resolved, but are thought to include those noted in Figure 1. Importantly, some of those mechanisms and resulting health benefits are strain-specific whereas others may be more widespread across probiotic strains (12). Therefore, it cannot be assumed that all probiotics will have the same effects. Products containing live microorganisms are also being developed and sold to support the health of extra-intestinal organs such as the vaginal-tract, lung, and skin. As a result, avast array of products containing live microorganisms including juices, diet bars, infant formulas, waters, chewing gum, sweeteners, pizza, toothpaste, and cosmetics are now available to consumers (16). These products are marketed toward individuals seeking to improve their mood, skincare, gut and vaginal health, and a myriad of other aspects of physical and psychological health and wellness, and many of these products claim to contain probiotics (17–19).

Dietary supplements, foods, and other probiotic-containing products are not regulated in the same manner as drugs, which require evidence of clinical efficacy for curing, treating, preventing or mitigating a disease, and do not require premarket review by the Federal Drug Administration (FDA). Rather, dietary supplements are permitted to make general “well-being” claims that do not require FDA approval. If a product labeled as a dietary supplement makes a claim involving the cure, treatment, prevention or mitigation of disease, that product is considered to be an unapproved drug and is subject to FDA action (20). The extent to which claims on commercial probiotic products are substantiated is not clear, but recent retail surveys found that only about 35% of probiotic supplements and 50% of probiotic foods could be clearly linked to any health benefit (21, 22).

Previous reviews have examined probiotic use in at-risk or health-compromised individuals, discussed regulatory questions, and attempted to refine the definition of probiotic products (16, 18, 19, 23, 24), but few have considered the potential benefit (or harm) of probiotic use in military personnel specifically (25). Given the growing presence of these products in the marketplace, the high use of dietary supplements by military personnel, and the potential for (and marketing of) probiotic products to benefit general health, there is likely to be increased interest by military personnel in using probiotics.

**Figure 2:**
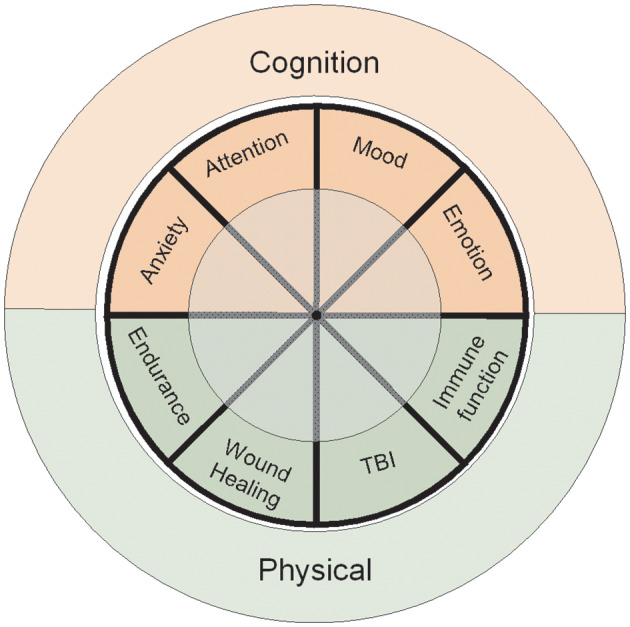
Primary focus areas of the review. The Cognitive Domain encompasses extensive literature review of human studies associated with memory, learning, and psychological states. The Physical Domain includes physical attributes, wound healing, traumatic brain injury (TBI), and host immunity. The review was conducted to assess the current body of evidence regarding the impact of probiotics directly relevant for military personnel performance and to identify key research gaps that must be addressed to establish probiotic efficacy within this population.

This review was conducted to assess the current body of evidence regarding the impact of probiotics in healthy adults on outcomes directly relevant to health and performance of military personnel, and to identify knowledge gaps where further research is needed to establish probiotic efficacy in this population. Given the broad scope and unique population, this review is intended to present a narrative overview of the evidence base with respect to military relevance. The review is organized using two health and performance core areas of importance to the military: physical and psychological domains, with specific sub-elements discussed for each domain, Figure 2.

## Search Criteria

Systematic search criteria were not used for this narrative review. However, to provide a comprehensive evaluation of the evidence base, authors conducted separate literature searches for each topic area included in the review using PubMed and/or Google Scholar. Searches used the logical operator “OR” between probiotic-related terms (e.g., “probiotic,” “Lactobacillus,” “Bifidobacterium,” “gut microbiome”) and the logical operator “AND” between the probiotic-related terms and topic-specific terms. For example, for the cognition topic area, either the cognition search modifier cogniti^*^ (i.e., “cognition,” “cognitive”), affective (i.e., “mood,” “emotion,” “anxiety,” “depression,” “stress”), or cognitive tasks (e.g., “stroop task”) were used. Reference lists of relevant narrative and systematic reviews were also manually searched. All searches were completed prior to September 2019; however, relevant studies published after that date were included if the authors were aware of their publication. Human intervention trials published in peer-reviewed literature were considered for inclusion irrespective of study design. Studies published solely in abstract form or in gray literature were not considered.

## Physical Domain

Exercise, especially of high-intensity or sustained for long periods of time, increases physiological stress, and metabolic demands (26). Those effects can induce transient oxidative stress, changes in intestinal permeability, and systemic inflammation (27). When recovery is insufficient, immune function can also be compromised. Probiotics have been proposed as a strategy for mitigating these effects through reduction of reactive oxygen/nitrogen species and inflammation, and for promoting intestinal barrier integrity and immune function (28–31). As such, multiple studies have examined whether probiotics and candidate probiotics can promote exercise performance, post-exercise recovery, and immune function during exercise training (32).

### Exercise Performance

Several studies have examined effects of various probiotics and candidate probiotics on exercise performance, and endurance performance in particular (Table 1). These studies have used both multi- and single-strain formulations, included tests of endurance, strength and power, and been conducted in a variety of populations including both endurance and skill athletes (33, 38, 40–42), and sedentary adults (37). Several have reported favorable effects. For example, increases in time to exhaustion have been reported with both multi-stain and single-strain products (35, 39), and, in one study, *Lactobacillus plantarum* PS128 supplementation reduced oxidative stress and improved performance during a triathlon (36). Confirmatory studies; however, are rare, and more often, studies have failed to demonstrate beneficial effects of probiotics on exercise performance. Indeed, a recent position stand on the use of probiotics in athletes concluded that while studies using multi-strain products seem to more often demonstrate benefit for aerobic performance than single-strain products, the majority of studies have found no effect on aerobic performance (32).

**Table 1:** Probiotic influence on physical performance in healthy individuals.

**References**	**Pop.^a^**	**Study design**	**Probiotic administration**	**Duration**	**Performance measures and results**
Carbuhn et al. (33)	16 collegiate swimmers; age not reported	DB, RCT	*B. longum* 35624 1 x 10^9^ CFU/d vs. placebo during intensified training period	6 weeks	Aerobic performance: no differences Anaerobic performance: no differences Lower body power: no differences Inflammation: no differences Immunity: no differences Cognitive stress: improved w/ probiotic
Cox et al. (34)	20 elite distance runners; 7 ± 6 years	DB, RCT	*L. fermentum* VRI-033 (PCC) 1.2 x 10^10^ CFU/d vs. placebo during training	4 months	Aerobic performance: no differences Illness: improved w/probiotic Inflammation: some markers improved
Huang et al. (35)	16 amateur runners 20–40 years	DB, RCT	*L. plantarum* TWK10. 1 × 10^11^ CFU/d vs. placebo	6 weeks	Aerobic performance: Increased run time to exhaustion Muscle damage (Creatine Kinase): no differences
Huang et al. (36) (study 1)	18 triathletes 19–24 years	DB, RCT	*L. plantarum* PS128 3 x 10^10^ CFU/d vs. placebo during triathlon training	4 weeks	Inflammation: improved w/probiotic Muscle damage: no differences
Huang et al. (36) (study 2)	16 triathletes 19–26 years	DB, RCT	*L. plantarum* PS128 3 x 10^10^ CFU/d vs. placebo during triathlon training	3 weeks	Lower body power: improved w/probiotic Aerobic performance: improved w/probiotic Inflammation: improved w/probiotic Muscle damage: some markers improved w/ probiotic Oxidative stress: improved w/probiotic
Ibrahim et al. (37)	21 sedentary young men; 21 ± 2 years	DB, RCT; parallel	*L. acidophilus* BCMC 12130, *L. casei* BCMC 12313, *L. lactis* BCMC 12451, *B. bifidum* BCMC 02290, *B. infantis* BCMC 02129, *B. longum* BCMC 02120 (6 x 10^10^ CFU/d) vs. placebo during circuit training program	12 weeks	Muscle strength and power: no differences Serum inflammation markers: no differences
Marshall et al. (38)	32 endurance runners 23–53 years		Multi-strain probiotic^b^ vs. multi-strain probiotic + glutamine^c^ vs. placebo during ultramarathon training	12weeks	Aerobic fitness: no differences Aerobic performance: no differences
Shing et al. (39)	10 runners 27 ± 2 years	DB, RCT	*L. acidophilus* (7 x 10^9^ CFU/d), *L. rhamnosus* (15 x 10^9^), *L. casei* (9 x 10^9^), *L. plantarum* (3 x 10^9^), *L. fermentum* (1 x 10^9^), *B. lactis* (4 x 10^9^), *B. breve* (1 x 10^9^), *B. bifidum* (0.5 x 10^9^), *S. thermophilus* (2 x 10^9^) vs. placebo	4 weeks	Aerobic performance: improved w/probiotic—increased run time to fatigue in the heat
Toohey et al. (40)	23 collegiate athletes 20 ± 1 years	DB, RCT	*B. subtilis* DE111 5 x 10^9^ CFU/d vs. placebo during resistance training program	10 weeks	Lower and upper body strength: no differences Lower body power: no differences Agility: no differences Body composition: improved w/probiotic
Townsend et al. (41)	25 collegiate baseball players 20 ± 1 years	DB, RCT	*B. subtilis* DE111 1 x 10^9^ CFU/d vs. placebo during offseason training	12 weeks	Lower body strength: no differences Lower body power: no differences Agility: no differences Anaerobic fitness: no differences Inflammation: improved w/probiotic Immunity: no differences Body composition: no differences
West et al. (42)	99 (35) cyclists 35 ± 9 years	DB, RCT	*L. fermentum* VR1-003 (PCC) 1 x 10^9^ CFU/d vs. placebo	11 weeks	Aerobic fitness: no differences Illness: improved w/probiotic in male, worsened w/probiotic in females Inflammation: improved with probiotic Immunity: no differences

*CFU, colony forming units; w/, with; /d, per day; DB, double-blind; RCT, randomized controlled trial*.

a *Mean ± SD; and/or range*.

b *L. acidophilus CUL-60 1 x 10^10^ CFU/d, L. acidophilus CUL-21 1 x 10^10^ CFU/d, B. bifidum CUL-20 9.5 x 10^9^ CFU/d, B. animalis subsp. lactis CUL-34 5 x 10^8^ CFU/d, 0.6 g fructooligosaccharide*.

c *L. acidophilus CUL-60 2 x 10^9^ CFU/d, L. acidophilus CUL-21 2 x 10^9^ CFU/d, B. bifidum CUL-20 5 x 10^10^ CFU/d, B. animalis subsp. lactis CUL-34 9.5 x 10^8^ CFU/d, L. salivarious CUL-61 5 x 10^9^ CFU/d, 0.9 g L-glutamine*.

*B., Bifidobacterium; L., Lactobacillus*.

### Exercise-Induced Muscle Damage and Recovery

Exhaustive and/or unaccustomed exercise induces temporary muscle damage resulting in delayed onset muscle soreness, loss of muscle strength and power, decreased muscle function, and impaired physical performance which require a complex cascade of mechanisms for repair and recovery (43). The combined effects of stress, inadequate rest and recovery, and suboptimal nutrition in military populations during and between exercise bouts can compromise or prolong recovery from exercise-induced muscle damage resulting in performance decrements and injury (44). Emerging evidence supports the possibility of a “gut microbiome-gut-muscle axis,” by which gut microbes influence muscle damage, growth, and repair through multiple interrelated mechanisms. These mechanisms are thought to include modulation of nutrient absorption, intestinal permeability, anabolic hormones (e.g., insulin-like growth factor-1), inflammation, immune function, and myocellular signaling (45–47). Accordingly, recent studies have begun to explore the efficacy of probiotics for reducing muscle damage and accelerating muscle repair and recovery (Table 2). Results of those studies have been largely inconclusive, but suggest some promise for specific probiotic strains. For example, Jager et al. reported that supplementation with *Bacillus coagulans* GBI-30, 6086 (BC30) and the protein casein (relative to casein alone) reduced soreness, attenuated increases in markers of muscle damage, and prevented a 5% decrease in some, but not all, measures of physical performance following a single bout of muscle damaging exercise in recreationally-active young men (49). However, the non-randomized, pre-post study design precluded determining whether the effects were due to BC30 supplementation or were simply a training effect. In a study of elite soldiers, BC30 in combination with β-hydroxy-β-methylbutyrate, prevented a 39% decrease in one of four measures of muscle integrity, but did not impact circulating markers of muscle damage or inflammation during a 40-d military training exercise compared to β-hydroxy-β-methylbutyrate alone (48, 51). In another study, daily supplementation with the combination of *Streptococcus thermophilus* FP4 and *Bifidobacterium breve* BR03 relative to placebo improved physical performance by ~10% without impacting perceived soreness, circulating markers of muscle damage, or muscle swelling following a muscle-damaging exercise bout in resistance-trained young men (50).

**Table 2:** Probiotic influence on exercise-induced muscle damage in adults.

**References**	**Pop.^a^**	**Study design**	**Probiotic administration**	**Duration**	**Measures and results**
Gepner et al. (48)	17 elite male soldiers; 20 ± 2 years	DB, RCT; parallel	*B.coagulans* GBI-30, 6086 (BC30; 1.0 x 10^9^ CFU/d) + CaHMB vs. CaHMB only during intense military training	40 days	Serum inflammation markers: no differences Serum muscle damage markers: no differences Muscle integrity: improved in rectus femoris; no differences in vastus lateralis
Jager et al. (49)	29 recreationally- trained men; 21 ± 3 years	SB, not random, pre-post design	*B. coagulans* GBI-30, 6086 (BC30; 1.0 x 10^9^ CFU/d) + casein vs. casein alone prior to muscle damaging exercise bout	2 weeks	Muscle soreness: improved w/ probiotic 72 h post-exercise but not 24–48 h Perceived recovery: improved w/probiotic Serum muscle damage markers:trend for improvement Muscle swelling: no differences Anaerobic power: trend for improvement Lower-body power: no differences
Jager et al. (50)	15 resistance-trained men; 25 ± 4 years	DB, RCT; crossover	*S. thermophilus* FP4 (5 x 10^9^ live cells/d), *B. breve* BR03 (5 x 10^9^ live cells/d) vs. placebo prior to muscle damaging exercise bout	3 weeks	Muscle soreness: no differences Muscle swelling: no differences Plasma inflammation markers: improved w/ probiotic at rest but not after exercise Plasma muscle damage markers: no differences Peak torque: improved w/ probiotic Range of motion: no differences

*CaHMB, calcium β-hyroxy-β-methylbutyrate; CFU, colony forming units; w/, with; /d, per day; DB, double-blind; RCT, randomized-controlled trial; SB, single-blind*.

a *Study population. Age is mean ± SD*.

*B., Bacillus; S., Streptococcus*.

### Training Stress and Respiratory Immunity

Several groups have published studies examining evidence concerning effects of probiotics on respiratory immune function in athletes. Recently, systematic reviews have been conducted on the evidence presented in these studies. King et al. reviewed 21 clinical trials involving 4,273 participants consuming *Lactobacillus* or *Bifidobacterium* probiotics (52). Similarly, Hao et al. performed a systematic review on 13 clinical trials, performing meta-analysis on 12 (3,270 participants) ranging from children to older adults, taking *Bifidobacterium, Lactobacillus*, and *Streptococcus* probiotics (13). Both groups concluded that the evidence presented supports probiotic benefit for reducing numbers of URTI episodes, duration, and related work absences (13, 52). However, Hao et al. raised concern over quality of evidence in their review, citing “low and very low quality of evidence” (13). Of particular relevance to this paper are those studies that focused on the severity and incidence of URTIs in healthy adults and athletes.

Intense training and exercise, especially when recovery is insufficient, can increase risk of immune impairment and URTIs (53–55). Following recurring periods of physical activity, which can be common in some military personnel, is a period termed the “open window” of immune suppression when it is suggested that pathogens are more likely to invade and establish infection (53). In fact, respiratory infectious diseases account for up to 30% of infection related military hospitalizations, and have been estimated to impact up to 80,000 recruits and 600,000 active duty service members each year (56). Ultimately, this has resulted in up to 27,000 lost training days and 95,241 lost duty days annually (56). Lost duty and training time can drastically reduce military strength and readiness.

Several studies show improvements in biomarkers or biochemistries associated with immune function following probiotic intervention. However, the overall evidence is mixed (Table 3). Seven studies have reported some benefit of probiotics on URTI symptoms, severity, or duration in athletes. Dose, probiotic strain, single- vs. multi-strain formulations, and duration (2–23 week) vary greatly across those studies. All studies showing some respiratory improvement included organisms from the genus *Lactobacillus*. What is lacking are associations between improvements in markers of immune function or respiratory illness to performance outcomes. In most studies, performance was not measured or no performance advantage was observed. The specific reasons are unknown, but this highlights challenges and opportunities for evaluating probiotics in the context of immune function and performance moving forward. In particular, there are few related studies conducted in military training environments which are often characterized by multiple stressors that can potentially compromise immunity including climate, intense physical training, sleep deprivation, suboptimal nutrition, and psychological pressures. One of the few published studies included 47 male French Commando cadets who spent most of their time in “heavy physical activities” and were categorized as “sleep deprived” over a ~4 week training event (76). The authors concluded that benefits of probiotic supplementation in a multi-stressor environment relied mainly on capacity to prevent the spread of infection throughout the respiratory tract; however, no differences in URTI were observed between the intervention and placebo groups.

**Table 3:** Impact of probiotics on GI and respiratory immunity in adults.

**References**	**Pop.^a^**	**Study design**	**Probiotic administration**	**Duration**	**GI or respiratory symptoms**	**Biochemistries**	**Performance outcome**
Clancy et al. (57)	17 male & 10 female recreational athletes; 16–40 years	PPI	2 x 10^10^ CFU/d *L. acidophilus* LAFTI L10	4weeks	Fatigued athletes present more episodes of URIs/year and lost more activities to illness	Fatigued athletes: increased IFN-gamma production by CD4 cells Non- fatigued athletes: increased salivary IFN-gamma	No performance comparison made between treatment groups.
Moreira et al. (58)	123 male & 16 female trained marathon runners; 39 ± 9 years	DB, PC, RCT, parallel	Milk based *L. rhamnosus* GG (LGG). 3 x 10^8^ CFU/mL. Participants drank 130 mL/day	3 monthes	No substantial difference in symptoms of atopy or asthma.	No difference between groups	No significant difference in marathon completion time between the treatment groups.
Tiollier et al. (59)	47 trained French Army cadets; 21 ± 0.4 years	DB, PC, RCT, parallel	Milk fermented by *L. casei* strain DN-114 001	3 weeks + 5 days	No difference between groups on ERTI in incidence.	Prevented the reduction of salivary IgA after training. Immune cells did not differ between groups. DHEA-S increased in probiotics group. Cortisol and prolactin did not change.	No performance comparisons made.
Kekkonen et al. (60)	123 male & 16 female trained marathon runners; 39 ± 9 years	DB, PC, RCT, parallel	Milk based *L. rhamnosus* GG (LGG). 3 x 10^8^ CFU/mL. Participants drank 130 mL/day	3 months	Decreased number (33%) and duration (57%) of GI symptoms 2 weeks after marathon, but no effects related to URS incidence, compared with placebo	Hematological parameters within reference range for both groups.	No significant difference in marathon completion time between the treatment groups.
Cox et al. (34)	20 elite male runners; 20–34 years	DB, PC	Capsules containing *L. fermentum*. VRI-003 (PCC) 12 x 10^9^ CFU/d	4 weeks	Reduction in number (50%) of days with respiratory illness symptoms (self-reported)	Modest increase in salivary IgA and IgA1, and IFN-γ. No change in IL-4 and IL-12.	No substantial changes in running performance measures
West et al. (42)	64 male 35 ± 9 years & 35 female 36 ± 9 years elite competitive cyclists	DB, PC, RCT, parallel	One capsule per day containing *L. fermentum*(PCC) 1 × 10^9^ CFU/d	11 weeks	Increase in mild GI and lower respiratory symptoms compared to placebo.	Reduced perturbations in anti-inflammatory and pro-inflammatory cytokines (IL-1RA, IL-6, IL-8, IL-10, GM-CSF, IFN-γ, TNF-α) in probiotic group.	No difference between groups in performance tests (cycle ergometry, VO_2_max) or exercise duration.
Martarelli et al. (61)	24 male recreational athletes; 32 ± 6 years	PC, RCT, parallel	Powdered mixtures of the 2 probiotic strains (1:1 *L. rhamnosus* IMC 501 and *Lactobacillus paracasei* IMC 502; ~10 × 10^9^ CFU/d)	4 weeks	NR	Increased plasma biological antioxidant potential in probiotic group.	No performance comparisons made between groups.
Gleeson et al. (62)	54 male & 30 female trained endurance athletes; 27 ± 11.6 years	DB, PC, RCT, parallel	Fermented milk containing *L. casei* Shirota 6.5 x 10^9^ CFU 2 times per day	16 weeks	Placebo group had 36% more URS and higher URTI episodes compared with probiotic group (1.2 vs. 2.1). Severity and duration of symptoms were not significantly different.	Salivary IgA concentration was higher after 8 and 16 weeks compared to placebo. No difference with IgG, IgM, or total immunoglobulin.	No performance comparisons made between groups.
Gleeson et al. (63)	66 trained endurance athletes; 19–28 years	DB, PC, RCT, parallel	Sachets containing L. salivarius^*^, 2 x 10^10^ CFU/d	16 weeks	No difference in URS duration between groups, no substantial difference in frequency, duration, or severity or URTI.	No difference in salivary IgA between groups. Probiotic group increased lymphocyte totals, no differences in other blood immune cells.	No performance comparisons made between groups.
Lamprecht et al. (64)	23 male trained athletes; 38 ± 5 years	DB, PC, RCT, parallel	Sachets containing *B. bifidum* W23 + *B. lactis* W51 + *E. faecium* W54 *+ L. acidophilus* W22 *+ L. brevis W63 + L. lactis* W58, 1 × 10^10^ CFU/d	14 weeks	NR	Reduced TNF concentration (25%) at rest and post-exercise, reduced exercise-induced protein oxidation (8%) compared to placebo. No difference in IL-6 production, or change in total oxidation status of lipids and malondialdehyde.	No performance comparisons made between groups.
Valimaki et al. (65)	125 male & 16 female trained runners; 40 years (22–69)	DB, PC, RCT, parallel	Milk based fruit drink with *L. rhamnosus* GG 4 × 10^10^ CFU/d	3 months	NR	Oxidized LDL lipids increased by 28% and 33% during the preparation period and decreased by 16% and 19% during the marathon run in the placebo and probiotic groups, respectively.	No performance comparisons were made.
West et al. (66)	241 male 35 ± 12 years & 224 female 36 ± 12 years trained runners	DB, PC, RCT, parallel	Sachets containing (i) *B. animalis* subsp. *lactis (*Bl-04), 2.0 × 10^9^ CFU/d (ii) *L. acidophilus* NCFM and *B. animalis* subsp. *lactis* Bi-07 (NCFM & Bi-07) 5 × 10^9^ CFU/d	164 days	A reduction in URTI episodes in probiotic groups. Symptom severity did not differ between groups.	NR	Significant decrease in activity intensity but increase in activity duration vs placebo.
Haywood et al. (67)	30 male elite rugby players; 20–28 years	PC, RCT, parallel	Capsules probiotics multi-species (L. gasseri^*^: 2.6 × 10^12^ CFU/d, B. bifidum^*^: 0.2 × 10^12^ CFU/d, and B. longum^*^: 0.2 × 10^12^ CFU/d)	4 weeks + 4 weeks washout	Decreased incidence and duration of URTI and GI illness compared to placebo. No difference in symptom severity.	NR	No performance comparisons were made.
Shing et al. (39)	10 male trained runners; 27 ± 2 years	DB, RCT, PC, cross-over	Capsule providing 7.4 x 10^9^ CFU/d of L. acidophilus^*^, 15.55 x 10^9^ CFU/d of L. rhamnosus^*^, 9.45 x 10^9^ CFU/day of L. casei^*^, 3.15 x 10^9^ CFU/d of L. plantarum^*^, 1.35 x 10^9^ CFU/d of L. fermentum^*^, 4.05 x 10^9^ CFU/d of B. lactis^*^, 1.35 x 10^9^ CFU/d of B. breve^*^, 0.45 x 10^9^ CFU/d of B. bifidum^*^, and 2.2 x 10^9^ CFU/d of S. thermophilus^*^	4 weeks + 3 weeks washout	Small reduction in symptoms of GI discomfort compared to placebo	A small-to-moderate reduction in urine lactulose:rhamnose. Significantly lower plasma LPS/GI permeability in probiotic group. No significant difference with IL-6, IL-10, and IL-1ra compared to placebo. No significant differences with hematological variables or urinary claudin-3 pre- vs. post- exercise.	Significant increase in running time to fatigue in high temperatures compared to placebo
O'Brien et al. (68)	67 recreational but untrained subjects; 18–35 years	PC, PPI	Fermented kefir beverage containing undefined *Lactobacillus^+^*1 x 10^9^CFU/serving, 2 servings/ week	15 weeks	NR	Plasma c-reactive protein (CRP) increased due to exercise, but no difference due to probiotic intervention.	No performance comparison made with respect to probiotic.
Gill et al. (69)	8 male trained adults; 26 ± 6 years	B, RCT, PC, cross-over	L. casei^*^ (1 × 10^11^ CFU/d)	1 weeks	NR	No significant changes in resting circulatory endotoxin concentration or plasma cytokine profile compared to placebo. Relative to pre-EHS concentrations, higher plasma concentrations of endotoxin TNF-α were observed compared to placebo.	No performance comparisons were made due to probiotic intervention.
Gleeson et al. (70)	156 male, 112 female recreational athletes; 21 ± 3 years	DB, PC, RCT, parallel	Fermented milk containing *L. casei* Shirota 6.5 x 10^9^ CFU/2 times per day	16 weeks	No differences related to URS, number of episodes, total symptom score, or episode duration.	Decreased IgG-specific antibodies for cytomegalovirus (CMV) and Epstein-Barr virus compared with baseline of probiotic group. No differences in immune cell counts.	No performance comparisons were made due to probiotic intervention
Michalickova et al. (71)	36 male, 14 female elite athletes; 18–28 years	DB, PC, RCT	Capsules containing *L. helveticus* LaftiL10 2 x 10^10^ CFU/d	14 weeks	Decrease in URTI episode duration and number of symptoms compared to placebo. No difference in symptom severity and incidence of URTI between groups.	No significant changes in leukocyte abundance, TBF-β serum levels, IL-10 from peripheral blood mononuclear cells (PBMCs), IFN-γ level from PBMCs or viability/proliferation of PBMCs upon antigen stimulation. Group effect for CD4+/CD8+ ratio was significant.	No performance comparisons were made due to probiotic intervention
Roberts et al. (72)	25 male, 5 female recreational triathletes; 35 ± 1 years	DB, PC, RCT	Capsule containing *L. acidophilus* (1 x 10^10^ CFU/d, *L. acidophilus* CUL-60 [NCIMB 30157] and 1 x 10^10^ CFU/d *L. acidophillus* CUL-21 [NCIMB 30156]), 16.8 mg/day *B. bifidum* and *lactis* (9.5 x 10^9^ CFU/d *B. bifidum* CUL-20 [NCIMB30172] and 5 x 10^8^ CFU/d *B. animalis* subsp. *lactis* CUL-34 [NCIMB 30153] + 55.8 mg/d fructooligosaccharide (FOS) with or without antixodants	12 weeks	GI symptom episodes were lower in the probiotic + FOS group at each month of prerace training, and the severity of GI symptoms was lower	Reduction in plasma endotoxin levels at pre-race and 6 days post-race, as well as for IgG levels recorded 6 d postrace. No significant difference in GI permeability between groups Lactose:Mannitol increased marginally from baseline to pre-race and 6 days post-race with probiotic +antioxidant.	Non-significant trend of faster overall time to finish in probiotic groups.
Strasser et al. (73)	13 male, 16 female trained athletes; 22–30 years	DB, PC, RCT, parallel	Sachet containing 1 x 10^10^ CFU multispecies *B. bifidum* W23 + *B. lactis*W51 *+ E. faecium*W54 *+ L. acidophilus*W22 *+ L. brevis* W63 *+ L. lactis*W58	3 months	Incidence of URTI decreased for both groups over 12 weeks, yet fewer probiotic treated subjects had URTI after 12 weeks (5 vs 8).	After the acute exercise, probiotic group lost less tryptophan vs. placebo. Female participants had higher degradation of tryptophan compared with male participants.	Significant increase in training hours per week and decreased resting energy expenditure compared to placebo.
Marshall et al. (38)	24 male, 6 female trained endurance athletes; 23–53 years	RIM, parallel	Capsules with or without glutamine contained 1 x 10^10^ CFU/d, *L. acidophilus* CUL-60 and 1 x 10^10^ CFU/d *L. acidophillus* CUL-21 16.8 mg/d B. bifidum^*^ and *lactis* (9.5 x 10^10^ CFU/d, *B. bifidum* and 0.5 x 10^10^ CFU/d *B. animalis* subspecies *lactis*, and 55.8 mg/d fructooligosaccharides (FOS)	12 weeks	NR	Blood eHSP72 was not different between nutritional groups (probiotic with or without glutamine).	Time to race completion was not different between groups.
Michalickova et al. (74)	22 male elite athletes; 20–24 years	DB, PC, RCT, parallel	Capsules containing *L. helveticus* Lafti L10 (2 x 10^10^ CFU/d)	14 weeks	NR	Decreased malondialdehyde (MDA), superoxide dismutase activity (SOD) serum paraoxonase (PON1) compared to placebo.	No performance comparisons were made due to probiotic administration.
Carbuhn et al. (33)	20 female elite swimmers; 19–23 years	DB, PC, RCT, parallel	Capsules containing *B. longum* 35624 1 x 10^9^ CFU/d	6 weeks	Mild improvement in RESTQ52-sport weekly self-regulation scores of stress. No URTI or URS measures.	No difference in panel of systemic inflammatory markers. Endotoxin (LPS) and LPS-binding protein (LPB) were not statistically different between groups. Small but significant decrease in the systemic cytokine marker IL-1ra within the probiotic group at mid-training found.	No significant difference between supplemented groups
Komano et al. (75)	51 male recreational athletes; 19–21 years	DB, PC, RCT, parallel	Capsules containing heat killed *Lactococcus lactis* JCM 5805 1 x 10^11^ CFU/d	13 days	Significant decrease in some respiratory symptoms and cumulative days of URTI, decreased fatigue accumulation compared to placebo.	CD86 as maturation marker on dendritic cell activity was significantly increased in the probiotic group at day 14.	No difference between training time between groups. No performance comparisons were made due to probiotic intervention.

*Updated and adapted from AR 40-501 (2), Davies et al. (27), Pyne et al. (28), Coqueiro et al. (29). B, blinded; CFU, colony forming units; DB, double-blind; eHSP, extracellular heat shock protein; EHS, exertional heat stress GI, gastrointestinal; NR, not reported; PC, placebo controlled, PPI, pre-post intervention; RCT, random controlled trial; RIM, randomized independent measures; TGF, transforming growth factor; URS, upper respiratory symptoms; URTI, upper respiratory tract infections, URI, upper respiratory infection*.

a *Mean ± SD; and/or range*.

* *Strain not reported*.

+ *Species/strain not reported*.

*B., Bifidobacterium; C., Clostridium; E., Enterococcus; L., Lactobacillus, S., Streptococcus*.

### Training Stress and Gastrointestinal Barrier Injury

The lining of the GI tract is both a physical and immunological barrier, acting to deter the translocation of potentially harmful bacteria, toxins, and antigens into the systemic circulation while maintaining a selective permeability to nutrients (77, 78). GI barrier injury can lead to translocation of antigens such as bacterial LPS from the gut lumen into circulation. The resulting inflammation may contribute to GI distress and dysbiosis, adversely impact nutrient status, cognition and physical performance, and increase susceptibility to illness, infection and chronic disease (78–80). Of note, recent studies have reported increased GI permeability in military personnel during various training exercises in association with systemic inflammation, GI distress, increased blood brain barrier permeability, and changes in mood state (81–83). Those observations have stimulated interest in identifying interventions to prevent GI injury and mitigate increases in GI permeability within military environments.

To date, few studies have examined the efficacy of probiotics for mitigating GI barrier injury in healthy adults experiencing acute GI injury (Table 4). Those that have used different methods for inducing GI barrier injury included both prolonged moderate-to-high intensity exercise in various environments and non-steroidal anti-inflammatory drug (NSAID) ingestion. Of note, both stressors are common in military personnel (44, 86). Some, but not all, of these studies have reported beneficial effects of probiotic supplementation on GI permeability or other markers of GI barrier damage [Table 4; (39, 64, 69, 72, 76, 85)]. Notably, the majority of studies reporting beneficial effects have used multi-strain formulations. For example, Lamprecht et al. reported that 14-week supplementation with a multi-strain probiotic preparation reduced fecal zonulin (an indicator of intestinal barrier permeability) concentrations, and attenuated post-exercise increases in some, but not all, markers of inflammation and oxidative stress in physically active men (64). Similarly, Roberts et al. reported that a multi-strain formulation prevented increases in intestinal permeability and reduced circulating endotoxin concentrations during recovery from a triathlon (72). In contrast, studies using single-strains generally have not reported beneficial effects. Rather, in one study, plasma endotoxin concentrations were elevated for 24 h following strenuous exercise in the heat following 7 days of administration of *L. casei* (unknown strain) relative to placebo, suggesting a detrimental effect (69). Of note, *Lactobacillus* spp. do not produce LPS, thus the increased endotoxin concentrations did not reflect translocation of the ingested probiotic into circulation.

**Table 4:** Probiotic influence on markers of gastrointestinal barrier integrity in healthy adults.

**References**	**Pop.^a^**	**Study design**	**Probiotic manipulation/GI stressor**	**Duration**	**Measures and results**
**Exercise-induced GI injury**					
Carbuhn et al. (33)	16 female collegiate swimmers; age not reported	DB, RCT; parallel	*B. longum* 35624 1 x 10^9^ CFU/d vs. placebo during intensified training period	6 weeks	Inflammation: no differences Plasma endotoxin: no differences
Gill et al. (69)	8 endurance trained male runners; 26 ± 6 years	DB, RCT; crossover	L. casei^*^ (10^11^ CFU/d) vs. placebo/ 2 h run at 60% max intensity in a hot environment	7 days	Plasma endotoxin (post-exercise): worsened w/ probiotic Inflammation (post-exercise): no differences
Lamprecht et al. (64)	23 endurance trained men; 38 ± 4 years	DB, RCT; parallel	*B. bifidum* W23, *B. lactis* W51, *E. faecium* W54, *L. acidophilus* W22, *L. brevis* W63, *L. lactis* W58 (10^10^ CFU/day) vs. placebo/ 90 min intense cycling	14 weeks	GI permeability (indirectly measured): improved w/ probiotic Inflammation: 1 of 2 markers improved w/ probiotic
Roberts et al. (72)	20 recreationally active adults; 35 ± 2 years	DB, RCT; parallel	*L. acidophilus* CUL-60 (10^9^ CFU/d), *L. acidophilus* CUL-21 (10^9^ CFU/d), *B. bifidum* CUL-20 (9.5 x 10^9^ CFU/d), *B. animalis* subsp *lactis* CUL-34 (5 x 10^8^ CFU/d), 0.4 g FOS vs. placebo/ Long-distance triathlon	13 weeks	Small intestinal permeability: improved w/ probiotic Plasma endotoxin: improved w/ probiotic
Shing et al. (39)	10 male runners; 27 ± 2 years	DB, RCT; crossover	*L. acidophilus* (7 x 10^9^ CFU/d), *L. rhamnosus* (16 x 10^9^ CFU/d), *L. casei* (3 x 10^9^ CFU/d), *L. plantarum* (3 x 10^9^ CFU/d), *L. fermentum* (10^9^ CFU/d), *B. lactis* (4 x 10^9^ CFU/d), *B. breve* (10^9^ CFU/d), *B. bifidum* (4.5 x 10^8^ CFU/d), *S. thermophilus* (2 x 10^9^ CFU/d) vs. placebo/ Run to fatigue at 80% max intensity in a hot environment	4 weeks	Gastroduodenal permeability: no differences Small intestinal permeability: no differences Serum LPS: no differences Inflammation: no differences
**NSAID-induced GI injury**					
Endo et al. (84)	29 chronic aspirin and omeprazole users + iron deficiency anemia; 72 ± 7 years	DB, RCT; parallel	L. casei^*^ (45 x 10^8^ to 62 x 10^9^ CFU/d) vs. placebo/ Chronic aspirin use	3 months	Mucosal damage, small intestine: improved w/ probiotic
Gotteland et al. (85)	16 healthy adults; 23 ± 4 years	DB, RCT; crossover	*L. rhamnosus* GG (2.4 x 10^9^ CFU/d), *L. helveticus* (2.4 x 10^9^ CFU/d), *L. acidophilus* (2.4 x 10^9^CFU/d) containing dairy product vs. same dairy product after heat treatment vs. no treatment/ Indomethacin ingestion	5 days	Gastroduodenal permeability: improved w/ probiotic Small intestinal permeability: no differences
Krumbeck et al. (76)	94 obese adults; 44 ± 11 years	DB, RCT; parallel	*B adolescentis* IVS-1 (10^9^ CFU/d) vs. *B. animalis* subsp *lactis* BB-12 (10^9^ CFU/d) vs. IVS-1+GOS vs. BB-12+GOS vs. GOS vs. lactose/ Aspirin ingestion	3 weeks	GI permeability: no differences Serum LPS: no differences

*CFU, colony forming units; DB, double-blind; FOS, fructo-oligosaccharides; GI, gastrointestinal; GOS, galacto-oligosaccharide; LPS, lipopolysaccharide; NSAID, non-steroidal anti-inflammatory drug; RCT, randomized-controlled trial; w/ = with*.

* *Strain not reported*.

a *Mean ± SD and/or range*.

*B., Bifidobacterium; C., Clostridium; E., Enterococcus; L., Lactobacillus, S., Streptococcus*.

### Physical Domain Summary

The ability of various different single-strain and multi-strain probiotic products to improve physical, primarily endurance, performance, often through effects on immunity, inflammation, and gut barrier integrity have been tested in athlete populations. Very few have been conducted in military populations. Within all of the physical performance related outcomes reviewed, some strains and strain-combinations have shown potential efficacy in single studies, but confirmatory studies are rare which precludes confident conclusions that any specific single- or multi-strain probiotic will benefit a particular outcome such as endurance performance or exercise-induced muscle damage. This may indicate strain-specific effects, but could also reflect heterogeneity in the populations studied, dosages used, and duration of trials among other factors.

The majority of probiotic studies conducted in athlete populations have focused on immunity (32), and the incidence and severity of URTIs in particular. Again, some strains show benefit, while others do not, and confirmatory trials are rare. However, the multiple studies showing benefits coupled with meta-analyses suggesting favorable effects of probiotics on URTI incidence and severity in non-athlete populations support the need for clinical trials in military personnel, particularly during prolonged training events. Similarly, several, but not all, strains and strain combinations have shown beneficial effects on GI barrier injury during exercise. Those studies, coupled with evidence that certain probiotic supplements (i.e., *Escherichia coli* Nissle1917 and VSL#3) improve symptomology in chronic GI diseases that are associated with barrier injury and increased permeability support the need for related research in military populations (19, 87, 88). Thus, definitive recommendations for or against the use of certain single- and multi-strain probiotic formulations for favorable influence on physical performance and related outcomes in military personnel cannot be made at present. However, positive effects of some products in athlete and non-athlete population underscores the need for probiotic research focused on physical performance outcomes and mediators in military populations, and identifies potential candidates for testing.

## Cognitive and Psychological Health Domain

Emerging evidence suggests a bidirectional relationship between intestinal microbiota and human brain function, termed the “gut-brain axis.” Intestinal microbes are thought to modulate this axis by altering the enteric nervous system and vagus nerve signaling, as well as immune function, and by producing compounds that enter systemic circulation and cross the blood brain barrier (89, 90). Probiotic intake has shown benefits in certain neurological disorders and may also ameliorate depressive and chronic fatigue syndrome, and anxiety symptoms (91, 92). In addition to emerging research on probiotics for psychological and neurological disorders, studies have also examined the influence on probiotic intake on cognitive function, mood, and emotional states in healthy individuals. Cognition, mood and emotion can be categorized into multiple sub-domains that are measured with a variety of different validated tests and scales (Tables 5, 7). Responses to these tests can vary along a continuum in healthy individuals, particularly in times of stress (93–95), and provide insight into effects of probiotics on cognition, mood and emotional state in healthy adults. Below we review studies assessing effects of probiotics within these sub-domains.

**Table 5:** Categorization of cognitive tasks.

**Cognitive domain**	**Cognitive tasks**
Motor Speed and Information Processing	Cogstate Detection Test Cogstate Groton Maze Chase Test Cogstate Identification Test Motor Screening Test
Attention	Rapid Visual Information Processing Task
Learning and Memory	Cogstate Groton Maze Final Recall Cogstate International Shopping List Test Cogstate One Card Learning Test Immediate and Delayed Recall Paired Associates Learning Wechsler Memory Scale
Cognitive Control	Attention Switching Task Cogstate One Back Test Digit Span Test Emotional Stroop Test Stroop Test Verbal Learning Test

### Probiotic Effects on Cognitive Control in Healthy Adults

Motor speed and information processing refers to the speed and accuracy of processing incoming information (96). Administration of single strains of *Lactobacillus* had marginal benefits in choice response time and social psychomotor performance but did not influence other measures of visuomotor speed, sensorimotor ability, or sustained attention (97, 98). Similarly, administration of *B. longum* did not influence sustained attention in another study (99). Together the findings provide little evidence of benefit of probiotic intake on motor speed, information processing, and attention, as summarized in Table 6.

**Table 6:** Probiotic influence on cognition in healthy individuals.

**References**	**Pop.^a^**	**Study design**	**Probiotic manipulation**	**Duration**	**Cognitive measures and results**
Allen et al. (99)	22 healthy adults, 25.5 ± 1.2 years	DB, RM	*B. longum* 1714 strain vs. placebo	4 weeks	Learning: Probiotic improved Sustained attention: No differences Emotion recognition: No differences Emotional Stroop: No differences
Benton et al. (100)	126 healthy adults, 48–79 61.8 ± 7.3 years	DB, RCT	65 mL L. casei^*^-containing (6.5 x 10^9^ CFU) vs. placebo milk	20 days	Short-term memory: Probiotic impaired after 20 (not 10) days Long-term memory: No differences Verbal fluency: No differences Intelligence: No differences
Chong et al. (101)	111stressed adults, 18–60 years	DB, RCT	*L. plantarum* DR7(1 x 10^9^ CFU) vs. placebo powder	12 weeks	Social Emotion Cognition: Probiotic improved speed Verbal learning and memory: Probiotic improved speed Psychomotor performance: No differences Attention: No differences Visual learning and memory: No differences Associate learning: No differences Working memory: No differences Executive function: No differences
Chung et al. (102)	36 healthy older adults, 60–75 65.0 ± 1.1 years	DB, RCT	*L. helveticus* IDCC3801 (500, 1,000, vs. 2,000 mg) vs. placebo capsules	12 weeks	Sustained Attention: 1,000 mg probiotic improved Selective attention: 500 mg probiotic improved Working memory: No differences Short-term memory: No differences Long-term memory: No differences
Kelly et al. (97)	29 healthy adults, 20–33 24.6 ± 0.8 years	DB, RCT, Cross-over	*L. rhamnosus* JB-1 (1 x 10^9^ CFU) vs. placebo capsules	4 weeks	Memory: No differences Attention switching: No differences Sustained Attention: No differences Emotional interference: No differences Emotion recognition: No differences
Lew et al. (98)	103 stressed adults, 18–60 years	DB, RCT	*L. plantarum* P8 (2 x 10^10^ CFU) vs. placebo sachets	12 weeks	Social emotional cognition: Probiotic improved speed Memory: Probiotic improved Target detection and identification: No differences
Papalini et al. (103)	58 healthy adults, 18–40 years	DB, RCT	Multispecies^b^ probiotic (5 x 10^9^ CFU) vs. placebo powder	4 weeks	Emotional reactivity: No differences Emotional interference: No differences Selective attention: No differences Pre vs. post stress working memory: Probiotic improved

*CFU, colony forming units; DB, double blind; RM, repeated; M, measures; RCT, randomized controlled trial*.

* *Strain not reported*.

a Mean ± SD and/or range

b *B. bifidum W23, B. lactis W51, B. lactis W52, L. acidophilus W37, L. brevis W63, L. casei W56, L. salivarius W24, L. lactis W19, and L. lactis W58*.

*B, Bifidobacterium; L, Lactobacillus*.

Learning and memory is perhaps the most widely studied cognitive domain within the probiotic literature. Learning refers to a change in behavior resulting from experience, and memory refers to retaining and retrieving that information. In individuals experiencing moderate life stress, *L. plantarum* P8 intake improved episodic memory, compared to placebo, but had no effects on other aspects of learning and memory, such as visual learning and semantic memory (98). In another study, four to 12 weeks of multi-strain probiotic supplementation did not influence visual or verbal learning and memory across multiple tests (97, 99, 101).

Episodic and working memory are cognitive domains most sensitive to decline with age, and thus older adults have been the primary focus in this area (104). Intake of a *L. casei* Shirota-containing milk drink worsened work memory compared to placebo after 20 days of consumption, and had no effect on episodic memory in one study of older adults (100). Twelve weeks of *L. helveticus* supplementation did not influence short- or long-term memory (102). Whether probiotics impact memory in healthy younger adults has not been studied.

Cognitive control, also called executive function, consists of mental set shifting (moving back and forth between tasks), information updating (integrating new information, also termed working memory), and inhibition (holding back a prepotent response) (105). In one comprehensive study of a multi-strain probiotic, supplementation did not influence selective attention, emotional interference or neural responses, but did improve working memory performance compared to placebo, following a stressor (103). These findings suggest that certain probiotics may ameliorate working memory deficits during stress (106, 107). In contrast, probiotics appear to exert fewer effects under non-stressful conditions. In support, of this observation single-strain *Lactobacillus* administration did not influence different measures of executive function or working memory in two studies (97, 101). Collectively, the existing evidence suggests that probiotic intake may exert benefits during stressful, rather than non-stressful, experiences. Further research should explore whether set-shifting and inhibition, in addition to information updating, are sensitive to probiotic improvements during stress.

### Probiotic Effects on Mood and Emotion, Depression, Anxiety, and Stress in Healthy Adults

Emotions are episodic, specific to a triggering event (108). Moods are longer lasting affective states, not necessarily linked to a triggering event (109) (Table 7). In one study of older adults, consuming a *L. casei* Shirota-containing milk drink resulted in a reduction of feelings of depression, but not alteration in mood state (100). Similarly, in another study, individuals suffering moderate life stress at baseline experienced reduced feelings of stress and anxiety using one measurement scale but not another, following *L. plantarum* P8 supplementation (98). Correlation analyses of the cognitive findings reported above showed that social emotion cognition, and verbal learning and memory improved after 12 weeks of probiotic intake and were associated with reductions in stress and anxiety. In a study of individuals experiencing moderate life stress at baseline, probiotic administration ameliorated feelings of stress and anxiety, reduced cortisol and proinflammatory cytokine levels, and increased anti-inflammatory cytokine levels (101). The results point to a potential relationship between probiotic-induced changes in mood, cognition, and the physiological stress response as seen in Table 8.

**Table 7:** Categorization of mood and emotion scales.

**Affect**	**Assessment**
Depression	Beck Depression Inventory Depression, Anxiety and Stress Scale Geriatric Depression Scale Hospital Anxiety and Depression Scale Leiden Index of Depression Sensitivity
Anxiety	Beck Anxiety Inventory Depression, Anxiety and Stress Scale Hospital Anxiety and Depression Scale State Trait Anxiety Inventory
Stress	Depression, Anxiety and Stress Scale Perceived Stress Scale
Discrete Mood Scales	Bond Lader Mood Scales Profile of Mood States Hopkins Symptom Checklist
Emotion Regulation	Coping Checklist Primary Appraisal/Secondary Appraisal Scale

**Table 8:** Probiotic influence on mood in healthy individuals.

**References**	**Pop.^a^**	**Study design**	**Probiotic administration**	**Duration**	**Mood measures and results**
Allen et al. (99)	22 healthy adults, 25.5 ± 1.2 years	DB, RM	*B. longum* 1714 strain vs. placebo	4 weeks	Stress: Probiotic reduced daily stress Anxiety: Probiotic reduced
Benton et al. (100)	126 healthy adults, 48–79 61.8 ± 7.3 years	DB, RCT	65 mL L. casei^*^-containing(6.5 x 10^9^ CFU) vs. placebo milk	~3 weeks	Elated/depressed: Probiotic reduced depression in lowest baseline tertile for depression only Energetic/ tired, clearheaded/muddled, composed/anxious, confident/ unsure, agreeable/angry: No differences
Chong et al. (101)	111 stressed adults, 18–60 years	DB, RCT	*L. plantarum* DR7(1 x 10^9^ CFU) vs. placebo powder	12 weeks	Stress: Probiotic reduced Anxiety: Probiotic reduced Depression: No differences
Chung et al. (102)	36 healthy older adults, 60–75 years	DB, RCT	*L. helveticus* IDCC3801 (500, 1,000, vs. 2,000 mg) vs. placebo capsules	12 weeks	Depression: No differences Stress: No differences
Kelly et al. (97)	29 healthy adults, 20–33 24.6 ± 0.8 years	DB, RCT, Cross-Over	*L. rhamnosus* JB-1 (1 x 10^9^ CFU) vs. placebo capsules	4 weeks	Depression: No differences Anxiety: No differences Stress: No differences
Lew et al. (98)	103 stressed adults, 18–60 years	DB, RCT	*L. plantarum* P8 (2 x 10^10^ CFU) vs. placebo sachets	12 weeks	Stress [DASS]: Probiotic reduced Anxiety: Probiotic reduced Stress [PSS]: No differences Depression: No differences
Marotta et al. (110)	38 healthy adults, 19–33 22.00 ± 3.02 years	DB, RCT	*L. fermentum* LF16, *L. rhamnosus* LR06, *L. plantarum* LP01, and *B. longum BL04* (4 x 10^9^ CFU)vs. placebo powder	6 weeks	Depression Sensitivity: No differences total score; Probiotic increased acceptance Anxiety: No differences Depression [BDI]: No differences Depression [POMS]: Probiotic reduced Anger/hostility: Probiotic reduced
Messaoudi et al. (111)	55 healthy adults, 30–60 Probiotic: 42.4 ± 7.5 years Placebo: 43.2 ± 8.5 years	DB, RCT	*L. helveticus* R0052 and *B. longum* R0175 (3 x 10^9^ CFU) vs. placebo stick	4 weeks	Anxiety: Probiotic reduced anxiety Depression [HSCL-90]: Probiotic improved depression Global Psychopathology Severity: Probiotic reduced severity Somatization: Probiotic reduced somatization Anger/hostility: Probiotic reduced anger/hostility Depression [HADS-D]: No differences Stress: No differences
Messaoudi et al. (112)^b^	25 healthy adults, 30–60 years	DB, RCT	*L. helveticus* R0052 and *B. longum* R0175 (3 x 10^9^ CFU) vs. placebo stick	4 weeks	Stress: Probiotic reduced Obsessive compulsive: Probiotic reduced Anxiety: Probiotic reduced Paranoid-ideation: Probiotic reduced
Noorwali et al. (113)^c^	60 healthy adults, 18–40 years	DB, RCT	*L. acidophilus* CUL60 and CUL21, *B. lactis* CUL34, and *B. bifidum* CUL20 vs. placebo capsules	6 weeks	Anxiety: No differences
Owen et al. (114)^b^	50 healthy adults, 19–38 32.2 ± 3.8 years	DB, RCT	*L. acidophilus* CUL60 and CUL21, *B. lactis* CUL34, and *B. bifidum*CUL20 (2.5 × 10^10^ CFU) vs. placebo capsules	6 weeks	Anxiety: Probiotic reduced Depression: No differences Stress: No differences
Papalini et al. (103)	58 healthy adults, 18–40 years	DB, RCT	Multispecies^d^ probiotic (5 x 10^9^ CFU) vs. placebo powder	4 weeks	Depression: No differences Depression sensitivity: No differences
Steenbergen et al. (115)	40 healthy adults, Probiotic: 20.2 ± 2.4 yr Placebo: 19.7 ± 1.7 yr	DB, RCT	Multispecies probiotic (>2.5 x 10^9^ CFU/g) vs. placebo powder	4 weeks	Cognitive reactivity to sad mood: Probiotic reduced Aggression: Probiotic reduced Rumination: Probiotic reduced Depression: No differences Anxiety: No differences
Wang et al. (116)	40 healthy adults, 18–50 years	DB, RCT	*B. longum* 1714 (1 x 10^9^ CFU) vs. placebo powder	4 weeks	Distress: No differences Mood: No differences Exclusion perception: No differences

*BDI, Beck Depression Inventory; CFU, colony forming unit; NR, not reported; DASS, Depression, Anxiety and Stress Scale (DASS-42); PSS, Perceived Stress Scale; POMS, Profile of Mood States; HSCL-90, Hopkins Symptom Checklist-90; HADS-D, Hospital Anxiety and Depression Scale-Depression, DB, double blind, RM, repeated measures, RCT, randomized controlled trial, TB, triple blind*.

* *Strain not reported*.

a *Mean±SD and/or range*.

b *Secondary analysis of Messaoudi et al. (111) in participants with lowest urinary free cortisol (UFC) levels at baseline*.

c *Conference Proceedings*.

d *B. bifidum W23, B. lactis W51, B. lactis W52, L. acidophilus W37, L. brevis W63, L. casei W56, L. salivarius W24, L. lactis (W19 and W58)*.

*B, Bifidobacterium; L, Lactobacillus*.

In individuals not characterized by elevated depression and stress, 30 days of supplementation with a multi-strain probiotic improved somatization, depression and anger–hostility symptoms, depression, and anxiety, without impacting biomarkers of stress (111). Probiotic intake also reduced participants' reliance on self-blame as a coping strategy for negative experiences (111). Secondary analyses were performed in participants characterized by lower initial stress with probiotic intake also improving stress and obsessive compulsive and paranoid-ideation symptoms (111). In other studies, *L. helveticus* IDC3801-containing milk did not influence stress or depression in healthy older adults (102), and *L. rhamnosus* supplementation did not influence depression, anxiety, or stress, coping strategies to negative experiences, or emotional responses to an acute stressor in young adults (97). In healthy volunteers, daily intake of *B. longum* 1714 attenuated cortisol output and subjective anxiety in response to stress, and reduced daily reported stress. Resting electroencephalography (EEG) showed that *B. longum* increased frontal midline mobility, indicative of prefrontal cortex activity, and decreased Cz-theta power, often associated with memory (99). The same strain did not influence emotional responses to a stressor involving social stress and exclusion in another study, but did influence brain activity as measured by magnetoencephalography (MEG) both during a resting state and following the social stressor. These results were interpreted to indicate that *B. longum* modulates neural oscillations in response to acute stress (116).

A number of studies have evaluated the influence of probiotic administration on cognitive reactivity to sad mood, in addition to sad moods themselves. Steenbergen and colleagues examined the influence of 4 weeks of administration of a multi-strain probiotic relative to placebo on cognitive reactivity to depressed mood (115). Probiotic administration reduced overall cognitive reactivity to depression, as well as cognitive reactivity to aggressive and ruminative thoughts specifically. Probiotic intake did not influence depression or anxiety (115). In other studies, 4 weeks of probiotic intake did not influence depression or cognitive reactivity to depressed mood (103), and 6 weeks of probiotic intake did not influence overall cognitive reactivity to depressed mood, but enhanced acceptance and coping of sad mood (110). Probiotic intake also reduced feelings of depression and anger, although not depression when assessed using an alternate measurement scale. Many of the studies described above also assessed the administration of probiotics on aspects of mood and emotion. The probiotics *L. casei* Shirota and *L. plantarum* P8 supplemented individually resulted in reduced negative mood; however, *L. helveticus* IDCC3801and *L. rhamnosus* (JB-1) individually failed to produce similar outcomes (97, 98, 100, 102). Similarly, when multi-strain probiotics were administered, favorable effects were seen in some studies but not others (111, 115, 117). Further, studies finding positive effects on some aspects of mood often failed to find effects on others, e.g., *L. plantarum* P8 reduced feelings of stress on one scale, but not on another scale, and did not reduce anxiety or depression (98). Of the 11 studies that examined whether probiotic administration benefits mood and emotion, seven found evidence of improvement (Table 8). Three studies found that probiotic administration reduced symptoms of depression and two studies found reduced cognitive reactivity to depressed mood (100, 110, 111, 115). Four studies found that probiotic administration reduced symptoms of anxiety (99, 101, 110, 111). Four studies found that probiotic intake reduced perceived stress, two of those in populations characterized by moderate stress and two in populations characterized by normal to low stress (98, 99, 101, 112).

In summary, studies investigating the influence of probiotics on mood and emotion have selected a range of bacterial strains from the *Lactobacillus* or *Bifidobacterium* genera. Studies employing *Lactobacillus* strains found reduced negative mood with some species (*casei* and *plantarum*) but not others (*helveticus and rhamnosus)* (97, 98, 100–102, 110). Similarly, two studies employing combinations of *Lactobacillus* and *Bifidobacterium* (*L. helveticus* and *B. longum*) found reduced negative mood while one (six strains of *Lactobacillus* and three strains of *Bifidobacterium*) did not (103, 111, 115). Thus, the research to date does not point to a genus- or strain-specific effect of probiotics on mood. Nevertheless, the current evidence, suggests that probiotic intake may improve mood, particularly depressed, anxious, or stressed moods in healthy individuals free from mood disorders. Although the mechanism is not understood, it may involve reductions in physiological markers of stress and inflammation, such as cortisol and proinflammatory cytokines.

## Bridging Physical and Psychological Domains: Wound Healing and TBI

Wound healing is an intricately orchestrated process comprised of the temporally sequenced, but overlapping stages of homeostasis, inflammation, proliferation, and remodeling (118, 119). Multiple factors common in military operational environments can disrupt wound healing. These include non-hygienic conditions, increased presence of pathogens and rate of infection, increased stress, immuno-compromise, heightened psychological stress, and suboptimal nutrition (118, 120, 121). In support, it has been noted that deployment length correlates with level of psychological stress, which in turn, has been recognized to slow wound healing processes (120, 122). Impaired wound healing represents a significant healthcare burden for military populations, and current therapies are not always effective, underpinning an interest in developing novel approaches to improving wound care (119). Additionally, future military conflicts will likely require more field-based medical care before the wounded can be evacuated, further stimulating interest in identifying novel approaches to wound care that will decrease fatalities.

Probiotics have garnered some attention as potential adjuvants to standard wound care. Accumulating evidence from animal and *in vitro* studies suggest that these agents could improve wound healing through multiple mechanisms. Both topically and orally administered probiotics have been effective in animal studies, with, effects of oral probiotic therapies on wound healing thought to be mediated by interactions between the GI microbiota, enteric and central nervous systems, immune system, and skin microbiota (123). Whether these findings translate to human cohorts is unclear (123, 124). The largest body of clinical evidence derives from surgical care literature wherein several meta-analyses have examined whether probiotics and/or synbiotics (a combination of probiotics and prebiotics) reduce the incidence of post-operative infections, which impair healing of surgical wounds. In a recent meta-analysis of 28 studies including 2,511 patients undergoing GI surgery, perioperative synbiotic and probiotic administration reduced the likelihood of post-operative wound infection by 49 and 35%, respectively (125). A separate meta-analysis including many of the same studies (31 total studies) and 2,952 patients undergoing elective abdominal surgery found that synbiotics and probiotics were more effective than placebo for reducing risk of post-operative surgical site infections (73 and 45% reduced risk, respectively) (126). Substantial heterogeneity across studies in the type, dose, timing of administration, and length of treatment (range: 3–25 days) was noted, although most studies used a combination of multiple probiotic strains (125, 126). *Lactobacillus* spp. comprised the most commonly investigated probiotics, and *Bifidobacterium* spp., *Clostridium butryricum, Enterococcus faecalis* T-110, *Leuconostoc mesenteroides* 77:1, *Pediacoccuspentosaceus*5-33:3, *Streptococcus thermophilus, Streptococcus faecalis*, and *Saccharomyces boulardii* were also used in one or more studies (126). In a study of older adults undergoing colorectal surgery, a probiotic cocktail containing *L. acidophilus* LA-5, *L. plantarum, B. lactis* BB-12, and *S. boulardii* administered 1day before and for 15 days after colorectal surgery significantly reduced the likelihood of post-operative wound infection (127). Few other clinical studies have examined the efficacy of probiotics for promoting wound healing [Table 9; (128–135)]. However, those studies have reported beneficial effects of orally-administered multi-strain probiotics on healing of chronic ulcers, and beneficial effects of topical single-strain products on healing of infected burns.

**Table 9:** Probiotic influence on wound healing in adults.

**References**	**Pop.^a^**	**Study design**	**Probiotic administration**	**Duration**	**Measures and results**
Blanchet-Rethore et al. (128)	21 adults w/ atopic dermatitis and carrying *S. aureus*; 33 ± 12 years	NB; non-random	Heat-treated *L. johnsonii* NCC 533 (HT La1) lotion 0.3% w/w twice daily vs. untreated contralateral lesion	3 weeks	*S. aureus* load: Improved w/probiotic
Gueniche et al. (129)	75 men/women w/ atopic dermatitis; 6–70 years	DB, RCT; parallel	Lotion containing 5% V. filiformis^*^ lysate vs. placebo lotion	30 days	Lesion severity: Improved w/ probiotic TEWL: no differences
Gueniche et al. (130)	62 women w/ sensitive skin; 32 ± 12 years	DB, RCT; parallel	*L. paracasei* NCC 2461 (ST11) 1 x 10^10^ CFU/d vs. placebo	8 weeks	TEWL: improved w/ probiotic
Lee et al. (131)	110 women w/ dry skin; 49 ± 4 years	DB, RCT; parallel	*L. plantarum* HY7714 1 x 10^10^ CFU/d vs. placebo	12 weeks	TEWL, face: improved/ probiotic TEWL, forearm: improved w/ probiotic TEWL, hand: no differences
Mohseni et al. (132)	60 adults w/ diabetic foot ulcer; 60 ± 10 years	DB, RCT; parallel	L. acidophilus, L. casei, L. fermentum, B. bifidum^*^(2 x 10^9^ CFU each/d) vs. placebo	12 weeks	Wound healing: improved w/ probiotic
Ogawa et al. (133)	118 adults w/ elevated TEWL; 41 ± 8 years	DB, RCT; parallel	Heat-killed *L. brevis* SBC8803 25 or 50 mg/d vs. placebo	12 weeks	TEWL, forearm: no differences TEWL, neck: no differences TEWL, face: no differences
Peral et al. (134)	80 adult burn patients; 18–55 year	^#^RCT;parallel	*L. plantarum* ATCC 10241 culture (1 mL/cm^2^ burn area vs. standard care)	10 days	Wound healing: improved by probiotics in infected 3rd degree burns, but not infected 2nd degree burns or non-infected 3rd degree burns.
Peral et al. (135)	34 adults w/ chronic leg ulcers; 40–70 years	NB, non-random;pre-post trial	*L. plantarum* ATCC 10241 culture (no placebo)	10–30 days	Wound healing: Total healing after 30 days in 43% of diabetics and 50% of non-diabetics.

*CFU, colony forming units; DB, double-blind; NB, not blinded; RCT, randomized controlled trial; TEWL, transepidermal water loss; w/ with*.

a *Population. Age is mean ± SD or range*.

* *Strain(s) not identified*.

# *Blinding not described*.

*B, Bifidobacterium; L, Lactobacillus*.

Several studies have examined effects of probiotics on skin barrier integrity, an integral component of wound healing that can be assessed by measuring transepidermal water loss (TEWL) (136). Because skin barrier damage increases water permeability through the skin, decreases in TEWL over time can be used to compare the efficacy of different interventions on wound healing (137). Studies using TEWL as an outcome have reported both positive and null results regarding the efficacy of probiotics for improving skin barrier integrity in individuals with various skin conditions ranging from sensitive skin to atopic dermatitis [Table 9; (129–131, 133)]. In one of the studies, a reduction in the colonization of the skin with *Staphylococcus aureus*, a common skin pathogen, was also observed using a topical lotion containing *Vitreoscilla filiformis* (129). However, to what extent those findings are applicable to wounds caused by trauma (e.g., laceration, burns) is uncertain.

Taken together, the current evidence base demonstrates a potential for probiotics, especially when paired with prebiotics (i.e., synbiotics), to reduce infection risk at post-operative wound sites relative to placebo which could improve post-operative wound healing. However, substantial heterogeneity in treatment strategies across studies currently prevents reaching conclusions on optimal strain selection, dosage, and timing of administration. Aside from studies conducted in patients undergoing elective surgery, there is currently little evidence to support or refute the use of probiotics or candidate probiotics for promoting wound healing in healthy adults (Table 9). Most, if not all, of the studies examining effects of probiotics on wound healing or related outcomes have been conducted in individuals with chronic health issues, and whether similar effects should be expected in healthy populations is unclear. Nonetheless, multiple plausible mechanisms by which probiotics could influence wound healing exist, and there is some support from both preclinical and clinical studies supporting efficacy (123–126, 138).

### Traumatic Brain Injury

From 2000 to 2018, nearly 400,000 U.S. Armed Forces service members were diagnosed with traumatic brain injury (TBI) (139). Notably, TBI and comorbidities such as post-traumatic stress disorder (PTSD) have also been associated with gut microbiota dysbiosis in animal models, suggesting more work in humans is warranted (140–142). These associations have stimulated interest in examining the role of the gut microbiome-gut-brain axis in the etiology and persistence of TBI-associated comorbidities, and in examining the effectiveness of probiotics as novel therapeutics in patients with a history of TBI (143–145).

A recent systematic review by Brenner et al. identified only two published human studies examining probiotic interventions for treatment of TBI and/or PTSD (143). In one of the studies, brain trauma patients requiring enteral feeding had fewer infections, shorter stays in the intensive care unit, and fewer days on mechanical ventilation when receiving a diet supplemented with glutamine and *Lactobacillus johnsonii* (La 1) compared to treatment with a standard diet (146). In a separate study, Tan et al. randomized patients with severe TBI receiving enteral nutrition to receive *B. longum, L. bulgaricus*, and *S. thermophilus* (strains not reported) in addition to standard treatment or standard treatment alone for 21 days (147). Patients receiving the probiotic intervention demonstrated an improved immune response, reduced inflammation, fewer infections, and shorter stays in the intensive care unit (147).

The results of those two studies are largely consistent with a recent meta-analysis of 30 trials which reported that using probiotics in the treatment of critical illness of varied etiologies, and *L. plantarum*-containing supplements in particular, was associated with a 20% reduction in infection risk (148). The TBI studies also provide preliminary evidence supporting beneficial effects of probiotics on clinical outcomes in the early stages of recovery from critical illness caused by severe brain injury. However, as was noted by Brenner et al., neither study included assessment of longer-term psychological outcomes following recovery from the initial brain injury (143). As such, the usefulness of probiotics in treating psychiatric comorbidities of TBI remains undetermined. Likewise, the extent to which the aforementioned findings relating to effects of probiotics on cognition and mood in healthy adults translate to improving cognitive and psychological function in individuals with a history of TBI is unclear. Nonetheless, the evidence derived from studies of probiotic use on cognitive and mood outcomes in adults without TBI does provide rationale for continued investigation into the efficacy of probiotics for treating cognitive and psychological symptoms in individuals with a history of TBI.

## Gaps and Considerations

Military personnel are required to maintain standards of physical and psychological performance throughout their military careers and in any environment. While many similarities exist between military personnel and “professional” athletes, professional athletes prepare and perform with a singular focus, whereas military personnel must incorporate a broader and more variable regime. Many similarities also exist between military personnel and healthy adults. However, military personnel often operate under combinations of stress and extreme environments rarely experienced by most adults. Recommendations for military personnel will therefore ideally be based on studies conducted with military personnel in the environments in which they operate. Unfortunately, to date, few studies have examined effects of probiotics in military personnel (48, 59). However, it would be imprudent to not consider military applications of research conducted in other populations when evaluating potential applications of probiotics in military populations and identifying candidate strains for testing.

This narrative review has considered a growing evidence base that has assessed performance within physical, cognitive, and psychological domains following probiotic supplementation in healthy athletes and non-athletes (Table 10). In the course of the review, several knowledge gaps relevant to transitioning available evidence to military populations, and considerations for making recommendations for military populations were identified:

1. Studies do not always evaluate cognitive, psychological, and physical performance endpoints that are relevant for military training and performance, and generally do not incorporate measures assessing both performance domains. Multi-disciplinary studies are needed to address the complex interactions between physical and psychological stress imposed on military personnel.
2. Strain specificity is important for several, if not all, outcomes. However, studies reporting favorable effects of individual formulations, particularly within the physical performance domain, generally have not been reproduced. Confirmatory studies, especially those providing mechanistic insight, are needed to draw more definitive conclusions with respect to cause and effect.
3. Little consideration has been given to optimizing the timing, duration, and dose of probiotic administration. Studies examining these issues would provide a powerful approach to confirming the efficacy of promising formulations. Studies focused on optimizing the duration and dosing of supplementation are particularly relevant to military populations who may not receive much advanced notice before being placed in physically and psychologically stressing environments.
4. Little consideration has been given to how individual variability may contribute to probiotic effectiveness. Differences in sex, age, fitness level, gut microbiota composition, lifestyle habits, and environmental exposures may all influence interactions between a probiotic and host. Understanding these differences could conceivably lead to opportunities for personalized probiotic recommendations, or, more likely, recommendations for certain populations or environments.
5. Probiotic formulations are particularly relevant in certain military environments. Many probiotic strains require refrigeration which may not always be available to military personnel, and will not maintain viability under the extreme conditions required for military ration development and storage. One alternative approach to consider for future research is to consider prebiotics, inactivated microbes, and/or postbiotics which refers to metabolites and other compounds produced by microorganisms (149).
6. While probiotics have a demonstrated long history of safe use, the safety of novel candidate strains cannot be assumed. Indeed, caution is recommended when using probiotics in immunocompromised individuals and critical care patients (23). Safety and efficacy is required to be demonstrated for the targeted population. Further, the safety of dietary supplements is also not always known, and inaccurate claims, labels and/or unspecified product ingredients have been found on the market (18, 150). Research using off-the shelf products must verify what is actually in the product prior to testing.
7. With the exception of a few studies conducted in military cohorts, the physical and psychological stressors studied are not fully representative of what military personnel experience. Future studies in military populations should replicate, as much as possible, environmental and occupational stressors in order to fully elucidate any benefits of probiotic supplementation.

**Table 10:** Summary of probiotics used in Physical and Cognitive performance studies.

**Physical performance**	**Cognitive performance**
*Bacillus coagulans* GBI-30	*B. animalis* subsp. *lactis*^a^
*B. animali*s subsp. lactis^a^	*B. bifidum* CUL-20^a^
*B. bifidum*^a^^*^	*B. bifidum* W23^a^
*B. bifidum* W23^a^	*B. lactis* CUL-34^a^
B. breve^*^	*B. lactis* W51^a^
*B. adolescentis* IVS-1	*B. lactis* W52^a^
B. infantis^*^	*B. longum* R0175^a^
B. lactis^*^	L. acidophilus^*^
*B. lactis* BB-12^a^	*L. acidophilus* CUL-21^a^
*B. lactis* W51^a^	*L. acidophilus* CUL-60^a^
B. longum^*^	*L. acidophilus* W37^a^
*B. longum* 35624	*L. brevis* W63^a^
*B. subtilis* DE110	*L. casei* Shirota
C. butryricum^*^	*L. casei*W56^a^
*E. coli* Nissle 1917	*L. helveticus* IDCC3801^a^
*E. faecalis* T-110	*L. helveticus* R0052^a^
*Enterococcus faecium* W54^a^	*L. lactis* W19^a^
L. acidophilus^*^	*L. lactis* W58^a^
*L. acidophilus* CUL-21^a^	*L. plantarum* P8
*L. acidophilus* CUL-60^a^	*L. rhamnosus JB-1*
*L. acidophilus* LA-5^a^	*L. salivarius* W24^a^
*L. acidophilus* W22^a^	
*L. brevis* W63^a^	
*L. bulgaricus*^a^	
L. casei^*^	
*L. dulbruecki* subsp *bulgaris*	
*L. fermentum*^a^^*^	
*L. fermentum*VRI-003	
L. helveticus^*^	
L. paracasei^*^	
L. plantarum^*^	
*L. plantarum* 299V	
*L. plantarum* ATCC10241	
*L. plantarum* PS128	
*L. plantarum* WCFS1	
*L. rhamnosus*^a^	
*L. rhamnosus* GG	
*Lactococcus lactis* W58^a^	
*Leuconostoc mesenteroides* 77:1	
*P. pentasaceus* 5-33:3	
*S. boulardii*^a^	
Streptococcus faecalis^*^	
Streptoccus thermophilus^*^	

Probiotics administered both alone and as part of a cocktail;

a *probiotics administered solely in a cocktail*.

* *Strain not reported*.

*B, Bifidobacterium; C, Clostridium; E, Escherichia; L, Lactobacillus, S., Saccharomyces*.

## Future Directions for Military Probiotic Research

Adequately powered, double-blind, randomized controlled trials conducted in military personnel exposed to the range and combinations of stressors in which military populations operate will be the gold standard for determining probiotic application for military personnel. Studies should use well-defined probiotic strains, consider dose, timing of administration, and product formulations, and determine the impact of inter-individual differences and environmental exposures on probiotic-host interactions. To expedite transition, studies should leverage the research reviewed herein by attempting to replicate and extend favorable effects observed in athlete or other populations. Care should be taken not to generalize positive or null results to all strains, populations or environments. To the extent possible, these studies should also aim to glean mechanistic insight into host-probiotic interactions to better establish causal relationships.

## Conclusion

A growing body of evidence has examined the effects of a range of probiotic products on physical and cognitive performance in healthy young adult populations, to include athlete populations (32). These studies are building on an evidence base that has demonstrated efficacy of probiotics in a variety of clinical applications ranging from treating symptoms of GI disorder to preventing post-operative infections. We conclude that there is currently not compelling evidence to demonstrate that probiotics globally improve human physical performance, cognition or mood in healthy adults, and military personnel in particular. As such, recommending probiotic use for military personnel is premature. Promising evidence for strain-specific effects, and perhaps more broad effects in the case of immune function, has been demonstrated in some studies. It should not be expected that all probiotics will have similar effects, or that individual probiotics will have favorable effects for all outcomes, within all populations, and across all environments. As such, the possibilities for research on different strain combinations, administered in different forms and doses to different populations in different environments are seemingly endless. While testing these combinations in military personnel would be ideal for translation of findings into specific recommendations, it is not conceivable. Therefore, to expedite transition, it is recommended that studies conducted in comparable civilian populations be used to inform design of confirmatory studies in military cohorts especially when plausible mechanistic evidence is available. Those studies must consider unique exposures and requirements of military personnel to include optimal product dosing, timing and formulation, and the effects of inter-individual and environmental variability.

## Author Contributions

JK, SL, and KR contributed to physical performance sections. GG contributed to cognitive sections. RA, JK, MG, KM, and JS contributed to introduction and consensus sections, manuscript content and organization. All authors contributed to literature review and manuscript editing.

## Conflict of Interest

The authors declare that the research was conducted in the absence of any commercial or financial relationships that could be construed as a potential conflict of interest.
